# Predictors of quality of life among community psychiatric patients in a peri-urban district of Gauteng province, South Africa

**DOI:** 10.4102/sajpsychiatry.v24i0.1289

**Published:** 2018-09-20

**Authors:** Dumakazi Mapatwana, Andrew Tomita, Jonathan Burns, Lesley Robertson

**Affiliations:** 1 Department of Psychiatry, University of KwaZulu-Natal, South Africa; 2 Department of Psychiatry, University of Exeter, United Kingdom; 3 Department of Psychiatry, University of the Witwatersrand, South Africa

## Abstract

**Introduction:**

Few studies on quality of life (QoL) in the mentally ill population of South Africa have been conducted, but none in community-dwelling individuals. This study examined the QoL of psychiatric patients at community mental health clinics in Gauteng province of South Africa.

**Methods:**

A cross-sectional interview-based study was conducted on 121 adult patients attending community psychiatric clinics. To reduce the impact of acute psychiatric symptoms on subjective QoL, only clinically stable patients were included. Instruments used included the World Health Organization Quality of Life BREF domains (i.e. physical health, psychological health, social relationships and environment), the Brief Psychiatric Rating Scale (BPRS) for severity of illness and a socio-demographic and clinical questionnaire.

**Results:**

Just under half of the sample rated their overall QoL as good or very good. The strongest predictor of a poor QoL in all four domains was residual psychiatric symptomatology. The most severe BPRS scores were for the symptoms of depression, anxiety and somatic concern. Perceived social support significantly predicted a better QoL in the psychological, social relationships and environmental domains.

**Conclusion:**

This study highlights the negative impact of residual psychiatric symptoms on subjective QoL, and the importance of social support and enhancing QoL. If better QoL is the goal of care, then our findings highlight the importance of managing residual symptoms and promoting social support.

